# Short‐term outcomes of a prospective multicenter phase II trial of total neoadjuvant therapy for locally advanced rectal cancer in Japan (ENSEMBLE‐1)

**DOI:** 10.1002/ags3.12715

**Published:** 2023-07-11

**Authors:** Yoshinori Kagawa, Jun Watanabe, Mamoru Uemura, Koji Ando, Akira Inoue, Koji Oba, Ichiro Takemasa, Eiji Oki

**Affiliations:** ^1^ Department of Gastroenterological Surgery Osaka General Medical Center Osaka Japan; ^2^ Department of Surgery, Gastroenterological Center Yokohama City University Medical Center Yokohama Japan; ^3^ Department of Gastroenterological Surgery Graduate School of Medicine, Osaka University Suita Japan; ^4^ Department of Surgery and Science, Graduate School of Medical Sciences Kyushu University Fukuoka Japan; ^5^ Department of Biostatistics, School of Public Health The University of Tokyo Tokyo Japan; ^6^ Department of Surgery, Surgical Oncology and Science Sapporo Medical University Sapporo Japan

**Keywords:** locally advanced rectal cancer, neoadjuvant chemotherapy, non‐operative management, pathological complete response, radiation therapy, total mesorectal excision, total neoadjuvant therapy

## Abstract

**Aim:**

To evaluate the feasibility and safety of total neoadjuvant therapy (TNT) in patients with locally advanced rectal cancer (LARC) in Japan.

**Methods:**

This prospective, multicenter, open‐label, single‐arm phase II trial was conducted at five institutions. The key eligibility criteria were age ≥ 20 years, LARC within 12 cm from the anal verge, and cT3‐4N0M0 or TanyN+M0 at the time of diagnosis that enabled curative resection. Preoperative short‐course radiation therapy (SCRT) 5 Gy × 5 days (total 25 Gy) + CAPOX (six courses) followed by total mesorectum excision (TME) was the treatment protocol. Non‐operative management (NOM) was allowed if clinical complete response (cCR) was obtained in the preoperative evaluation. The primary endpoint was the pathological complete response (pCR) rate.

**Results:**

Thirty patients (male, *n* = 26; female, *n* = 4; median age, 62.5 [44–74] years; cT [T2, *n* = 1; T3, *n* = 25; T4, *n* = 4]; cN [N0, *n* = 13; N1, *n* = 13; N2, *n* = 4]) were enrolled. The final analysis included 30 patients in total. The completion rates were 100% for SCRT and 83% for CAPOX. TME and NOM were performed in 20 and seven patients, respectively. pCR was observed in six patients (30% [95% CI 14.0%–50.8%]). The primary endpoint was met. pCR+cCR was observed in 13 (43.3%) patients. There were no treatment‐related deaths. Grade ≥3 (CTCAE ver. 5.0) adverse events (≥20%), including diarrhea (23.3%) and neutropenia (23.3%). The median follow‐up period was 15.6 (10.5–22.8) months, with no recurrence or regrowth in NOM.

**Conclusions:**

ENSEMBLE‐1 demonstrated satisfactory pCR and cCR, and well‐tolerated safety of TNT for patients with LARC in Japan.

## Graphical abstract

This is the first phase 2 clinical trial conducted by a multicenter to investigate the feasibility and safety of total neoadjuvant therapy (TNT) for patients with locally advanced rectal cancer (LARC) in Japan. Pathological complete response (pCR) and pCR + cCR were observed in 30% and 43.3% of patients, respectively.

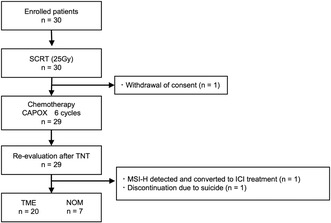

## 1 INTRODUCTION

Colorectal cancer (CRC) is the third most frequently diagnosed cancer and second leading cause of cancer‐related deaths worldwide. In 2021, 732 210 newly diagnosed cases of rectal cancer and 339 022 related deaths were reported worldwide.^1^

The outcomes of locally advanced rectal cancer (LARC) have been improved using multimodal treatment strategies. For local control of LARC, preoperative chemoradiotherapy (CRT) for LARC has been developed in Europe and the United States,^2^ while lateral lymph node dissection (LLND) for LARC has been developed in Japan.^3^ However, over the past decade, mortality rates have not improved with preoperative CRT or LLND because LARC is associated with a high rate of distant metastasis (29%–39%).^4^, ^5^

In recent years, total neoadjuvant therapy (TNT), in which preoperative chemotherapy and (chemo)radiotherapy are administered sequentially to patients with LARC, has been developed in Europe, the United States, and Asia to improve the long‐term prognosis of LARC. In contrast, the standard treatment for LARC in the Japanese guidelines is upfront surgery and adjuvant chemotherapy (ACT) with LLND as an option.^6^ To date, there have been no reports of prospective, multicenter clinical trials of TNT in patients with LARC in Japan.

Therefore, we conducted a prospective multicenter single‐arm ENSEMBLE‐1 trial to evaluate the feasibility and safety of TNT in Japanese patients with LARC.

## 2 PATIENTS AND METHODS

### 2.1 Trial design and participants

**FIGURE 1:**
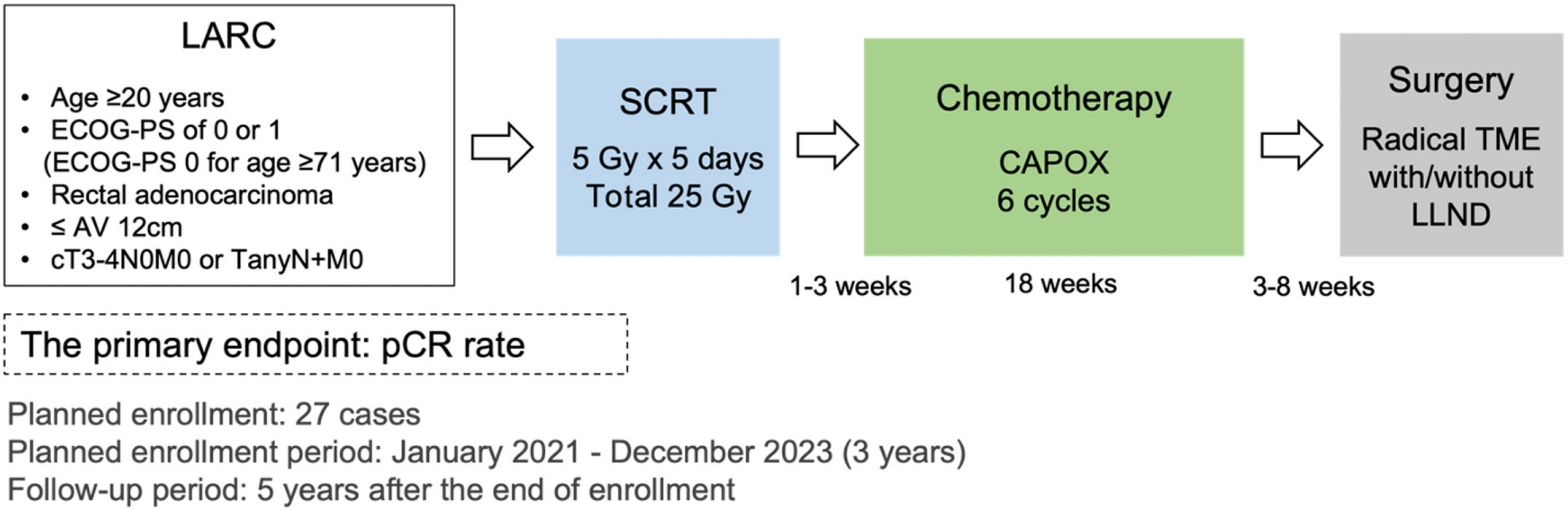
Study design. AV, anal verge; LARC, locally advanced rectal cancer; LLND, lateral lymph node dissection; pCR, pathological complete response; SCRT, short‐course radiotherapy; TME, total mesorectal excision.

The ENSEMBLE‐1 study was a prospective, multicenter, open‐label, single‐arm, phase II trial conducted at five institutions (Figure 1). The study protocol was approved by the Clinical Research Review Board of Osaka General Medical Center (ID: CRB5200005) and the institutional review board of each participating hospital before the initiation of the study. All patients provided written informed consent before enrollment in the study. This study was registered in the Japan Registry of Clinical Trials (jRCT s051200113).

The eligibility criteria were as follows: (1) written informed consent obtained; (2) a histological diagnosis of primary rectal adenocarcinoma; (3) no distant metastases on computed tomography (CT) or positron emission tomography (PET), and radical resection was clinically possible; (4) age ≥ 20 years; (5) Eastern Cooperative Oncology Group Performance status (ECOG‐PS) of 0 or 1 (ECOG‐PS 0 for age ≥ 71 years); (6) no prior treatment for rectal cancer; (7) lower margin of the tumor was within 12 cm from the anal verge (AV); (8) clinically diagnosed as Union for International Cancer Control (UICC) TNM classification (8th edition),^7^ cT3‐4 N0 M0 or Tany N+ M0; and (9) preserved organ function. The exclusion criteria were as follows: (1) patients undergoing major surgery, radiation therapy, or prior chemotherapy within 4 weeks of study inclusion; (2) a history of severe lung disease; (3) patients with a stent for stenosis; (4) HBs antigen or HCV antibody positivity; (5) serious comorbidities (heart failure, renal failure, liver failure, hemorrhagic peptic ulcer, intestinal paralysis, intestinal obstruction, uncontrolled diabetes, etc.); (6) active multiple cancers (simultaneous multiple cancers or metachronous multiple cancers with a disease‐free period of ≤5 years); and (7) pregnancy or breastfeeding. The complete inclusion and exclusion criteria are provided in Data S1.

### 2.2 Treatment

After registration, the patient received short‐course radiation therapy (SCRT) (5 Gy × 5 days; total 25 Gy), using three‐dimensional conformal radiation therapy or intensity‐modulated radiation therapy [IMRT], six cycles of CAPOX (capecitabine 2000 mg/m^2^ orally twice daily on days 1–14, oxaliplatin 130 mg/m^2^ intravenously on day 1, every 3 weeks), followed by total mesorectal excision (TME) or tumor‐specific mesorectal excision (TSME). The protocol stipulated that CAPOX should be initiated 14 ± 3 days after completion of SCRT. If treatment could not be started due to an adverse event, it could be delayed for up to 35 days. Surgery should be performed 3–8 weeks after the last dose of CAPOX (the last day of capecitabine administration) or the date of discontinuation. The following procedures were acceptable: low anterior resection (LAR), intersphincteric resection (ISR), abdominoperineal resection (APR), and Hartmann operation. In cases where invasion of adjacent organs is suspected, combined resection of adjacent organs was also acceptable to achieve radical resection. Additional LLND was acceptable at the discretion of the surgeon. The surgical approach (laparotomy, laparoscopy, or robot‐assisted surgery) was not specified.

Each patient underwent tumor restaging to complete clinical response (cCR), near CR (nCR), and incomplete CR (iCR), based on colonoscopy, pelvic magnetic resonance imaging (MRI), and digital findings according to Memorial Sloan Kettering Cancer Center (MSKCC) Regression Schema^8^ within 1–3 weeks from the completion of CAPOX (last day of capecitabine administration), or the date of discontinuation at each of the participating institutions. The clinical response rate based on pelvic MRI was also assessed using Response Evaluation Criteria in Solid Tumors (RECIST) v1.1. Non‐operative management (NOM) was allowed if a cCR was obtained in preoperative restaging, and the patient requested NOM.

### 2.3 Pathological analysis

Standard pathological analyses were performed on all resected specimens at each participating institution. The pathological primary tumor response to TNT was evaluated using the grading scale according to the Japanese Classification of Colorectal, Appendiceal, and Anal Carcinoma.^9^ Briefly, grade 0 represents no response to treatment; grade 1a, tumor size reduction of 1/3; grade 1b, tumor size reduction of 1/3 to 2/3; grade 2, tumor size reduction of >2/3; and grade 3, complete tumor ablation. Grade 3 corresponds to a pathological complete response (pCR).

### 2.4 Follow‐up

Follow‐up was performed every 3 months for the first 3 years and every 6 months thereafter for up to 5 years. Tumor markers carcinoembryonic antigen (CEA) and carbohydrate antigen 19–9 (CA19‐9) were assessed at each follow‐up examination. Chest‐abdominal‐pelvic computed tomography (CT) was performed every 6 months. Total colonoscopies were performed annually. For patients with NOM, tumor markers, colonoscopy, rectal examination, pelvic MRI, and chest‐abdominal‐pelvic CT every 4 months were recommended in the first 2 years and every 6 months thereafter for up to 5 years.

### 2.5 Endpoints and statistical analysis

The primary endpoint was the pCR rate. The secondary endpoints were R0 resection rate and safety in terms of adverse events, relapse‐free survival, overall survival, and recurrence pattern (local recurrence rate and distant recurrence rate).

The pCR rate of previous phase III trials in which SCRT was followed by surgical treatment was 0.5%–1%. With reference to these results and the fact that TNT will be added in this trial, we decided to examine whether 5% can be rejected in this trial. The expected pCR rate was set to 28% for patients treated with TNT followed by surgery as the expected value of the study treatment. With a pCR rate threshold of 5%, expected value of 28%, one‐sided significance level of 5%, and power of at least 80%, the sample size required by the exact binomial distribution method would be ≥19 patients. Based on the above, the expected total number of patients was set to 22. Considering the few dropouts, ineligible cases, and patients in whom resection was not performed, the target sample size was set to 27. The statistical setting was designed by a clinical statistician. The primary analysis population was a full analysis set (FAS) in which at least one dose of protocol therapy was administered, all selection criteria were met, no exclusion criteria were violated, and some data were available.

The pCR rate was estimated for the FAS, and 90% exact binomial confidence bounds were calculated. The overall frequencies and percentages were summarized for the demographic and clinicopathological characteristics. All statistical analyses were performed using SAS version 9.4 (SAS Institute Inc.) and GraphPad Prism version 6.01 for Windows (GraphPad Software).

## 3 RESULTS

### 3.1 Patient characteristics

**FIGURE 2:**
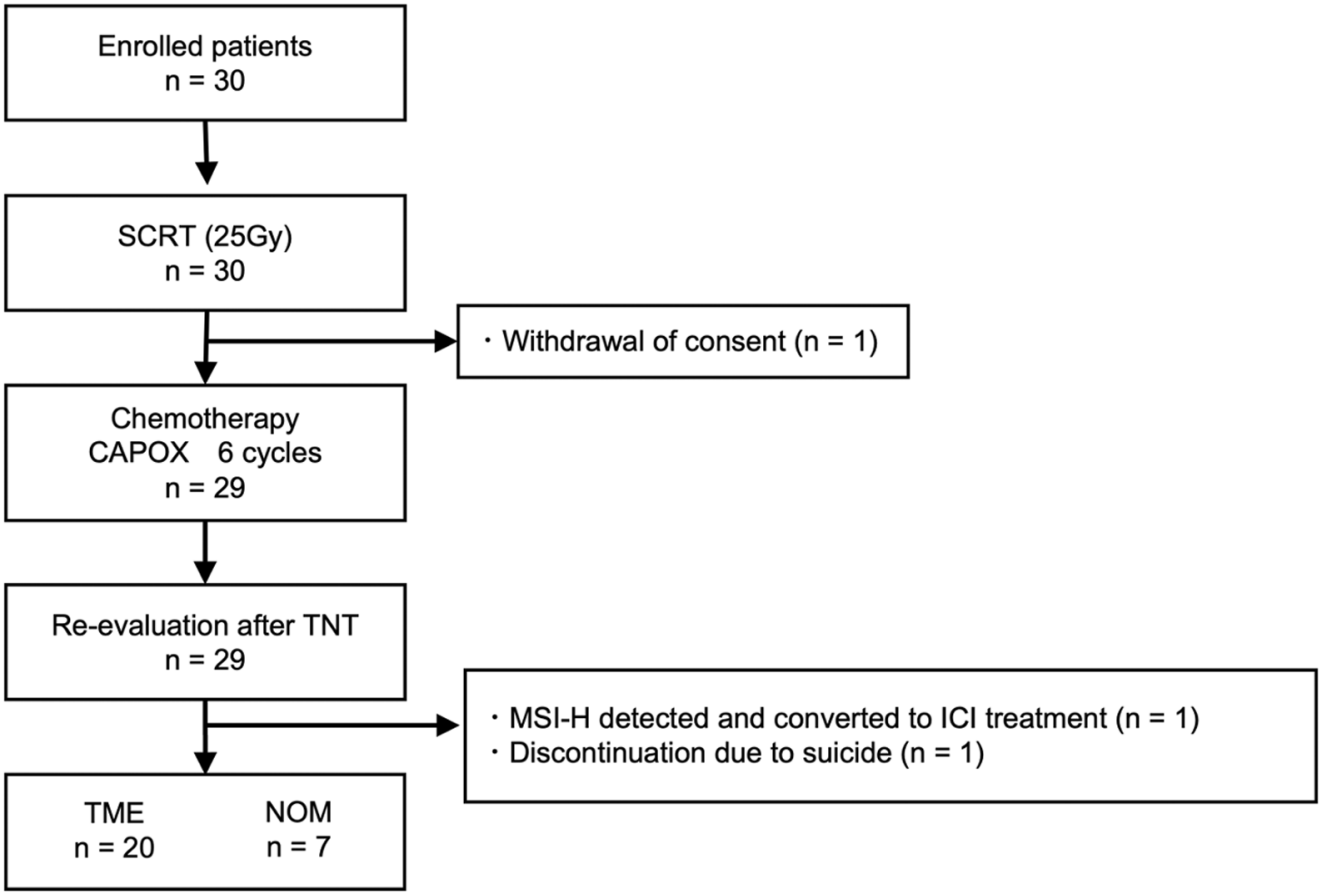
Patient consort diagram. Thirty patients were enrolled in this study. One patient withdrew their consent. One patient with an MSI‐high tumor experienced stable disease after TNT and converted the treatment to ICI. One patient committed suicide after the TNT evaluation. Among the other 27 patients, TME was performed in 20 patients, whereas NOM was performed in seven patients. ICI, immune checkpoint inhibitor; MSI, microsatellite instability; NOM, non‐operative management; SCRT, short‐course radiotherapy; TME, total mesorectal excision; TNT, total neoadjuvant treatment.

Thirty patients with LARC managed at five institutions between January 2021 and January 2022 were enrolled in this study. A flow diagram showing patient enrollment and progression through the study protocol is shown in Figure 2.

The clinical characteristics of the 30 enrolled patients are summarized in Table 1. The median age was 62.5 years (range, 44–74 years), and 26 patients (86.7%) were men. The pretreatment CEA concentrations ranged from 1.0 to 110 ng/mL (median 4.0 ng/mL). The median distance from the tumor to the anal verge was 60 mm (range: 0–120 mm). The median tumor diameter was 40 mm (range: 14–65 mm). The depth of the tumor was cT2 in one patient (3.3%), cT3 in 25 patients (83.3%), cT4a in three patients (10%), and cT4b in one patient (3.3%). Lymph node metastasis was classified as cN0 in 13 patients (43.3%), cN1 in 13 (43.3%), and cN2 in four (13.3%). The UICC TNM stage was cStage II in 12 patients (40.0%) and cStage III in 18 patients (60.0%).

**TABLE 1:** Patient characteristics.

Variables	*n* = 30
Age (years) (range)	62.5 (44–74)
Sex	
Male/Female	26 (86.7%)/4 (13.3%)
BMI (kg/m^2^) (range)	23.4 (16.5–32.2)
Pretreatment CEA (ng/mL) (range)	4 (1–110)
Pretreatment Hb (g/dL) (range)	13 (9–16)
Tumor size (mm) (range)	40 (14–65)
Tumor location AV (cm) (range)	6.0 (0–12.0)
Tumor location	
Ra/Rb	11 (36.7%)/19 (63.3%)
cT (UICC 8th edition)	
cT1	0
cT2	1 (3.3%)
cT3	25 (83.3%)
cT4a	3 (10.0%)
cT4b	1 (3.3%)
cN (UICC 8th edition)	
cN0	13 (43.3%)
cN1	13 (43.3%)
cN2	4 (13.3%)
Genomic status	
*RAS* wild/mutant/unknown	10 (33.3%)/19 (63.3%)/1 (3.3%)
*BRAF* wild/mutant/unknown	29 (96.7%)/0 (0.0%)/1 (3.3%)
MSS/MSI‐High/unknown	27 (90.0%)/2 (6.7%)/1 (3.3%)

*Note*: Data are presented as median (range) or number (%).

Abbreviations: BMI, body mass index; CEA, carcinoembryonic antigen; Hb, hemoglobin; AV, anal verge; MSS, microsatellite stable; MSI, microsatellite instability.

### 3.2 TNT

All patients received and completed the SCRT. The median treatment duration was 5 days (range; 5–9 days). There were no discontinuations due to adverse events. Twenty‐nine patients received CAPOX at a median of 14 days (range: 7–27 days) after the completion of radiotherapy. Chemotherapy initiation was postponed due to adverse events associated with radiotherapy in four patients (diarrhea). One patient withdrew their consent before receiving neoadjuvant chemotherapy. Twenty‐five patients (83.3%) completed six cycles of CAPOX. TNT was completed in 25 patients (83.3%) (Table 2).

**TABLE 2:** Outcomes of preoperative treatment and surgery.

TNT outcomes	*n* = 30
Completion of SCRT	30 (100%)
Completion of CAPOX	25 (83.3%)
Completion of TNT	25 (83.3%)
Re‐evaluation after TNT	29 (96.7%)
Clinical response to treatment (RECIST v1.1)	
cCR/cPR/cSD/cPD/NE	13 (43.3%)/14 (46.7%)/2 (6.7%)/0 (0.0%)/1 (3.3%)
Re‐evaluation (MSKCC regression Shema)	
cCR/nCR/iCR/NE	13 (43.3%)/13 (43.3%)/3 (10.0%)/1 (3.3%)
Treatment after TNT	
Surgical resection	20 (66.7%)
Non‐operative management	7 (23.3%)
Others^§^	3 (10.0%)

Surgical outcomes	*n* = 20
LAR/ISR/APR or Hartman	15 (75%)/3 (15%)/2 (10%)
With LLND/with diverting stoma	8 (40%)/17 (85%)
Operation time (min) (range)	336 (181–753)
Estimated blood loss (mL) (range)	29 (0–1000)
Conversion to open surgery	0 (0.0%)
Curative resection (Cur A)	20 (100%)
Number of lymph nodes resected (range)	14.5 (5–26)
Postoperative complication (Clavien‐Dindo Classification)	
Lower limb edema (grade I)	1 (5%)
Abdominal abscess (grade II)	1 (5%)
Liver failure (grade II)	1 (5%)
Urinary dysfunction (grade II)	1 (5%)
Paralytic ileus (grade IIIa)	1 (5%)
Postoperative hospital stay (days) (range)	13 (6–24)
Postoperative mortality	0 (0.0%)
Postoperative recurrence	0 (0.0%)

### 3.3 Restaging after TNT

Twenty‐nine patients (96.7%) were re‐evaluated for efficacy using MRI, colonoscopy, and a digital examination. MRI was performed at a median of 12 days (range, 0–29 days) after the last chemotherapy dose or the decision to discontinue chemotherapy, and a median of 154 days (range, 133–198 days) after the start of radiation therapy. The clinical response rates were as follows: cCR 43.3% (13/30), nCR 43.3% (13/30), iCR 10.0% (3/30), and not evaluable (NE) 3.3% (1/30) (Table 2). No distant metastases were detected during or after TNT.

### 3.4 Treatment after TNT

Seven of the 30 patients underwent NOM (23.3%), whereas 20 patients underwent TME (66.7%) (Table 2). One patient with iCR was found to have MSI‐H at restaging, and treatment was converted to ICI. The surgical approach was robotic in 19 cases and laparoscopic in one case. No conversion to open surgery was performed. Minimally invasive surgery was performed in all patients (100%). LAR, ISR, APR, and Hartman's procedure were performed in 15 (75%), three (15%), and two (10%) patients, respectively. LLND was performed in eight patients. Diverting stoma was created in 17 patients. Curative resection was performed in all 20 patients. Only one patient with a grade ≥3 complications (according to the Clavien–Dindo classification) was observed; the patient developed paralytic ileus. The median postoperative hospital stay was 13 days (range: 6–24 days). As of December 2022 (data cutoff), the median follow‐up time from enrollment was 15.6 months (range, 10.5–22.8 m). None of the patients who underwent both NOM and TME developed local recurrence or distant metastases.

### 3.5 Pathological findings

pCR, the primary endpoint, was achieved in six patients (6/20) (30% [95% CI 14.0%–50.8%]), and the final classification was ypStage 0 in six patients (30%), ypStage I in five patients (25%), ypStage II in five patients (25%), and ypStage III in four patients (20%) (Table 3). The downstaging rate in terms of T stage was 70.0% (14/20). Sixty percent (12/20) of the cT4 tumors were downstaged after TNT. The downstaging rate in terms of N stage was 50% (10/20). The proximal, distal, and radical margin were negative in all case. The histological therapeutic effect according to the Japanese Classification of Colorectal, Appendiceal, and Anal Carcinoma^9^ was classified as Grade 1 in 40% of cases, Grade 2 in 30%, and Grade 3 in 30.0%.

**TABLE 3:** Pathological findings.

Variables	*n* = 20
Histological type	
tub1/tub2/no findings	4 (20%)/10 (50%)/6 (30%)
Macroscopic classification	
Type 2/3/5/no findings	5 (25%)/5 (25%)/4 (20%)/6 (30%)
Histological therapeutic effect^a^	
Grade 1/2/3	8 (40%)/6 (30%)/6 (30%)
ypT	
ypT0	6 (30%)
ypT1	1 (5.0%)
ypT2	4 (20%)
ypT3	9 (45%)
ypT4	0 (0.0%)
ypN	
ypN0	16 (80%)
ypN1	4 (20%)
ypN2	0 (0.0%)
pCR	6 (30%)
Positive lymphatic invasion (ly)	4 (20%)
Positive vascular invasion (v)	7 (35%)
Positive tumor budding (BD)	15 (75%)
Positive neural invasion (Pn)	5 (25%)
Positive extramural cancer deposits (EX)	1 (5.0%)
Positive proximal margin (PM)	0 (0.0%)
Positive distal margin (DM)	0 (0.0%)
Positive radical margin (RM)	0 (0.0%)

*Note*: Data are presented as median (range) or number (%).

Abbreviations: tub1, well‐differentiated tubular adenocarcinoma; tub2, moderately differentiated tubular adenocarcinoma, pCR; pathological complete response.

^a^ According to the Japanese Classification of Colorectal, Appendiceal, and Anal Carcinoma by Japanese Society for Cancer of the Colon and Rectum.

### 3.6 Adverse events

Adverse events related to SCRT and CAPOX are shown in Table 4. The most common SCRT‐related adverse event was diarrhea (10.0%). The CAPOX‐related grade 3/4 adverse event rates, with an incidence rate of >10%, were as follows: diarrhea (23.3%), neutropenia (23.3%), and peripheral neuropathy (13.3%). One patient committed suicide after evaluation of the TNT and before surgery.

**TABLE 4:** Adverse events (CTCAE Ver.5.0).

Adverse events in TNT *n* = 30	Grade 1	Grade 2	Grade 3	Grade 4
Adverse events during SCRT				
Diarrhea	0 (0.0%)	1 (3.3%)	2 (6.7%)	0 (0.0%)
Adverse events during CAPOX				
Nausea/vomiting	9 (30.0%)	3 (10.0%)	0 (0.0%)	0 (0.0%)
Anorexia	7 (23.3%)	6 (20.0%)	1 (3.3%	0 (0.0%)
Diarrhea	7 (23.3%)	5 (16.7%)	6 (20.0%)	1 (3.3%)
Constipation	3 (10.0%)	2 (6.7%)	0 (0.0%)	1 (3.3%)
Elevation of AST or ALT	5 (16.7%)	2 (6.7%)	0 (0.0%)	0 (0.0%)
Elevation of amylase or γGTP	4 (13.3%)	0 (0.0%)	1 (3.3%)	0 (0.0%)
Fatigue	10 (33.3%)	8 (26.7%)	1 (3.3%)	0 (0.0%)
Hand foot syndrome	7 (23.3%)	4 (13.3%)	2 (6.7%)	0 (0.0%)
Peripheral neuropathy	14 (46.7%)	7 (23.3%)	4 (13.3%)	0 (0.0%)
Skin disorders	7 (23.3%)	0 (0.0%)	1 (3.3%)	0 (0.0%)
Neutropenia	2 (6.7%)	4 (13.3%)	5 (16.7%)	2 (6.7%)
Thrombocytopenia	6 (20.0%)	1 (3.3%)	2 (6.7%)	0 (0.0%)
Anemia	2 (6.7%)	2 (6.7%)	0 (0.0%)	0 (0.0%)
Renal dysfunction	0 (0.0%)	1 (3.3%)	0 (0.0%)	0 (0.0%)
Stomatitis or taste disorders	5 (16.7%)	0 (0.0%)	0 (0.0%)	0 (0.0%)
Treatment‐related death	0 (0.0%)			

*Note*: Data are presented as number (%).

Abbreviations: SCRT, short‐course radiotherapy; AST, aspartate aminotransferase; ALT, alanine aminotransferase; γGTP, gamma glutamyl transpeptidase.

## 4 DISCUSSION

This is the first phase II clinical trial conducted as a multicenter study to investigate the feasibility and safety of TNT in patients with LARC in Japan.

Over the last four decades, the outcomes of LARC have improved owing to significant advances in diagnostic imaging, surgical techniques, external radiation, and systemic chemotherapy. The recurrence rate of LARC in patients treated with surgery alone has improved with the standard performance of TME.^10^ Consensus management guidelines from the United States, Europe, and Japan have included TME and adjuvant chemotherapy as essential components of standard management for patients with LARC.^6^, ^11^, ^12^ To improve recurrence‐free survival and OS, the United States and Europe added CRT to TME and adjuvant chemotherapy^2^; on the other hand, Japan added LLND to TME.^3^, ^4^ Although these two approaches could decrease local recurrence, they did not show a significant impact on DFS or OS. Subsequently, to address this issue, clinical trials of TNT were conducted in the United States, Europe, and Asia and showed improvements in DFS and OS in phase III.^13^, ^14^, ^15^ In this study, eight patients (40%) underwent LLND after TNT. In this series, there was no metastasis to the lateral lymph nodes (LLN) after TNT, and the procedure was performed safely with no postoperative complications. There are no clinical trials that have investigated the efficacy and safety for LLND after TNT. LLND after CRT has been reported in retrospective studies to control local recurrence and improve long‐term outcome when performed in patients with preoperative LLN disease.^16^, ^17^ Even after TNT, LLND should be considered when LLN enlargements are present before treatment.

TNT consistently improved the pCR rate, approximately 20%–30%, including previous phase II trials.^13^, ^14^, ^15^, ^18^, ^19^ In this study, the pCR rate was 30%, and the pCR + NOM rate was 43.4% (Table 2), as NOM was allowed if cCR was obtained. In the OPRA trial, the cCR and nCR rates after TNT were 42.4% and 38.4%, respectively, and the 3‐year organ preservation rate was 79% in patients with cCR and 52% in patients with nCR.^20^ Currently, the improvement in pCR or cCR rates by TNT has received some recognition.

TNT enhances the chances of attaining pCR, which has traditionally been shown to correspond to higher OS and DFS.^21^ A meta‐analysis of TNT in comparison to standard care suggested that the pCR rate was improved, although the effect of TNT on OS and DFS was unclear.^22^ In randomized phase III trials of TNT reported to date, the RAPIDO^15^ and PRODIGE‐23^14^ trials reported significant improvements in distant failure and disease‐free survival (DFS), and the STELLAR^13^ trial reported a significant improvement in overall survival (OS) (Table 5). There is a lack of evidence regarding the optimal regimen for TNT, such as whether SCRT or long‐course CRT should be chosen, and whether triplet therapy should be added to chemotherapy. Further evaluation in prospective randomized trials is required.

**TABLE 5:** Summary of phase 3 trials of TNT.

	RAPIDO^15^		PRODIGE‐23^14^		STELLAR^13^	
Object	cT4a/T4b, cN2, MRF+, EMVI+, LLN+		cT3/cT4		T3‐4 and/or N1‐2	
Treatment	TNT	CRT	TNT	CRT	TNT	CRT
	RT [25Gy/5Fr] → CAPOX ×6/FOLFOX4 × 9 → TME	CRT [50–50.4Gy/25‐28Fr + Cape] → TME → CAPOX ×8/FOLFOX4 × 12	mFOLFIRINOX ×6 → CRT [50Gy/25F + Cape] → TME → mFOLFOX6 × 6/Cape ×4	CRT [50Gy/25F + Cape] → TME → mFOLFOX6 × 12/Cape ×8	RT [25Gy/5Fr] → CAPOX x 4 → TME ± CAPOX x 2	CRT [50Gy/25Fr + Cape] → TME ± CAPOX x 6
Case number	462	450	231	230	298	293
pCR	28%	14%	27.8%	12.1%	22.5%	12.6%
Local recurrence	8.3% (3y)	6.0% (3y)	4.8% (3y)	7.0% (3y)	8.5% (3y)	11.1% (3y)
DFS	23.7% (3y)*	30.4% (3y)*	76% (3y)	69% (3y)	64.5% (3y)	62.3% (3y)
OS	89.1% (3y)	88.8% (3y)	90.8% (3y)	87.7% (3y)	86.5% (3y)	71.5% (3y)

Abbreviations: CRT, chemoradiation therapy; DFS, disease free survival; EMVI, extramural venous invasion; LARC, locally advanced rectal cancer; MRF, mesorectal fascia; OS, overall survival; pCR, pathological complete response; TME, total mesorectal excision; TNT, total neoadjuvant therapy.

* Disease‐related treatment failure.

We demonstrated that SCRT followed by six cycles of CAPOX was well‐tolerated by patients, with a compliance rate of 83.3% (Table 2). Similarly, other RCTs reported favorable compliance rates of approximately 85%, with similar toxicities during neoadjuvant chemotherapy and radiotherapy. In the RAPIDO study,^15^ the incidence rate of grade ≥3 diarrhea was 17%, whereas it was 23.3% in the present trial (Table 4). In contrast, in STELLAR^13^ and PRODIGE‐23,^14^ the incidence rates of diarrhea were 6.4% and 6%, respectively. Therefore, attention to diarrhea is warranted in the RAPIDO regimen.

In the present trial, the rate of grade ≥3 postoperative complications was 5% and no treatment‐related deaths were observed. In previous phase III trials,^13^, ^14^, ^15^ the rate of postoperative complications was approximately 14% for TNT and CRT, while the rate of postoperative complications in patients who received TME + LLND was 22%.^3^

The present study had some limitations. Only a small number of patients were included in this study. Second, the follow‐up period was short because this trial was not designed to investigate long‐term prognostic improvement but rather to investigate the feasibility and safety of introducing TNT in the RAPIDO regimen in Japan. Third, although the circumferential resection margin is reported as a prognostic factor after rectal cancer surgery, we did not collect the date in this study for the same reason as above. Forth, the safety of NOM after TNT remains unclear. Careful follow‐up of patients is required in this trial.

A phase III clinical trial is currently ongoing in Japan to investigate the efficacy of SCRT followed by CAPOXIRI in comparison with SCRT followed by CAPOX in TNT, as well as the safety and efficacy of the NOM strategy for cCR or nCR in the ENSEMBLE trial (jRCTs031220342/NCT05646511).

In conclusion, this trial provided evidence that SCRT followed by six cycles of CAPOX for patients with LARC was feasible and well‐tolerated in Japanese patients with LARC. The long‐term efficacy of DFS and OS requires further exploration in future randomized trials.

## AUTHOR CONTRIBUTIONS

Authors made substantial contributions to conception and design, acquisition of data, and/or analysis and interpretation of data: Yoshinori Kagawa, Jun Watanabe, Mamoru Uemura, Koji Ando, Akira Inoue, Ichiro Takemasa, Koji Oba, Eiji Oki. Authors participating in drafting the article or revising it critically for important intellectual content: Yoshinori Kagawa, Jun Watanabe, Mamoru Uemura, Koji Ando, Akira Inoue, Ichiro Takemasa, Koji Oba, Eiji Oki. Authors giving final approval of the version to be published: Yoshinori Kagawa, Jun Watanabe, Mamoru Uemura, Koji Ando, Akira Inoue, Ichiro Takemasa, Koji Oba, Eiji Oki.

## FUNDING INFORMATION

None.

## CONFLICT OF INTEREST STATEMENT

The authors declare no conflicts of interest related to this study, except for Eiji Oki's conflict of interest regarding Chugai Pharmaceutical. The authors Jun Watanabe, Ichiro Takemasa, and Eiji Oki are editorial members of the *Annals of Gastroenterological Surgery*.

## ETHICAL APPROVAL AND CONSENT TO PARTICIPATE

The ENSEMBLE‐1 trials were conducted in accordance with the Declaration of Helsinki, the Japanese Ethical Guidelines for Medical and Health Research Involving Human Subjects, and the Clinical Trial Acts in Japan. Each trial was approved by the Osaka General Medical Center Certified Review Board. Written informed consent was obtained from all patients before enrollment.

## Supporting information


Data S1:

## Data Availability

Dr. Kagawa has full access to all data in the study and takes responsibility for the integrity of the data and accuracy of the data analysis.
