# Supplementary material for: Short‐term outcomes of a prospective multicenter phase II trial of total neoadjuvant therapy for locally advanced rectal cancer in Japan (ENSEMBLE‐1)
Source: Ann Gastroenterol Surg. 2023 Jul 11;7(6):968–76. doi: 10.1002/ags3.12715 (PMC10623965; doi:10.1002/ags3.12715)
Supplement: Supplementary file 1 — Data S1: [file AGS3-7-968-s001.docx]

**Supplement 1: Inclusion/Exclusion Criteria**

**Inclusion Criteria**

1. The patient was fully informed of the contents of the study and written informed consent was obtained.

2. The patient had a histologically confirmed rectal adenocarcinoma.

3. No distant metastasis on imaging studies was clinically amenable to curative resection.

4. Age ≥20 years on the date of consent

5. Eastern Cooperative Oncology Group Performance Status (ECOG PS) 0 to 1 (ECOG PS 0 if the patient was ≥71 years of age on the date of consent).

6. Prior untreated rectal cancer with inferior margin of the tumor within 12 cm of the anal verge (AV).

7. Clinically diagnosed as Union for International Cancer Control (UICC) TNM classification (8th edition) cT3-4N0M0 or Tany N+M0 at the time of diagnosis, prior to the start of treatment (a lymph node with a short diameter of ≥10 mm is considered positive).

8. The following criteria for major organ function are fulfilled within 14 days prior to enrollment. If more than one test result exists within the relevant period, the most recent result is used for the measurement. No blood transfusions or hematopoietic factor products should be administered within 14 days prior to the test date.

a. Neutrophil count: ≥1,500/mm^3^

b. Platelet count: ≥10.0 × 10^4^/mm^3^

c. Hemoglobin concentration: ≥9.0 g/dL

d. Total bilirubin: ≤1.5 times the upper limit of the institutional standard

e. AST, ALT, ALP: ≤2.5 times the upper limit of the institutional standard (≤5 times if the patient has liver metastasis)

Serum creatinine: ≤1.5 times the upper limit of the institutional standard, or creatinine clearance ≥45 mL/min.

**Exclusion Criteria**

1. Patients received any of the following treatments within a certain period prior to the start of the protocol:

a. Extensive surgery within 4 weeks (excluding CV port placement and stoma creation)

b. Any anticancer therapy within 4 weeks

c. Radiation within 4 weeks

2. Concomitant or preexisting severe pulmonary disease (interstitial pneumonia, pulmonary fibrosis, severe emphysema, etc.)

3. Patients with implanted colorectal stents

4. Patients with serious comorbidities (heart failure, renal failure, liver failure, bleeding peptic ulcer, intestinal paralysis, bowel obstruction, poorly controlled diabetes, etc.)

5. Patients with active multiple overlapping cancers (synchronous multiple overlapping cancers or iatrogenic multiple overlapping cancers with a disease-free period ≤5 years). However, carcinoma in situ (intraepithelial carcinoma) or intramucosal carcinoma that was considered curable by local treatment was not considered active multiple overlapping carcinoma.

6. Pregnant or lactating women, positive pregnancy test or unwillingness to use contraception

7. HBs antigen or HCV antibody positivity

8. Known human immunodeficiency virus (HIV) infection

9. Judged by the principal investigator or sub-investigator as unsuitable for this study.
